# Frequency transformation in the auditory lemniscal thalamocortical system

**DOI:** 10.3389/fncir.2014.00075

**Published:** 2014-07-08

**Authors:** Kazuo Imaizumi, Charles C. Lee

**Affiliations:** Department of Comparative Biomedical Sciences, Louisiana State University, School of Veterinary MedicineBaton Rouge, LA, USA

**Keywords:** tonotopy, receptive field, tracer injections, laser-scanning photostimulation, calcium imaging, optogenetics, whole-cell recording, brain slice

## Abstract

The auditory lemniscal thalamocortical (TC) pathway conveys information from the ventral division of the medial geniculate body to the primary auditory cortex (A1). Although their general topographic organization has been well characterized, functional transformations at the lemniscal TC synapse still remain incompletely codified, largely due to the need for integration of functional anatomical results with the variability observed with various animal models and experimental techniques. In this review, we discuss these issues with classical approaches, such as *in vivo* extracellular recordings and tracer injections to physiologically identified areas in A1, and then compare these studies with modern approaches, such as *in vivo* two-photon calcium imaging, *in vivo* whole-cell recordings, optogenetic methods, and *in vitro* methods using slice preparations. A surprising finding from a comparison of classical and modern approaches is the similar degree of convergence from thalamic neurons to single A1 neurons and clusters of A1 neurons, although, thalamic convergence to single A1 neurons is more restricted from areas within putative thalamic frequency lamina. These comparisons suggest that frequency convergence from thalamic input to A1 is functionally limited. Finally, we consider synaptic organization of TC projections and future directions for research.

The auditory thalamus, the medial geniculate body (MGB), receives ascending information from subthalamic stations and subsequently conveys it to the auditory cortex through thalamocortical (TC) projections (Imig and Morel, 1983; Anderson et al., 2006; Lee and Winer, 2011). At this stage, sound frequency information is transformed into separate concurrent pathways in different auditory cortical fields (Lee et al., 2004a). A variety of animal models and experimental techniques have contributed to an evolving understanding of these transformations. In this review, we integrate these past findings, current issues, and our recent data towards a unified understanding of sound frequency transformations in the auditory TC system. A more detailed review of anatomical and physiological studies in TC transformations is available elsewhere (Imaizumi and Lee, 2013).

## Principles of thalamocortical projections in the auditory lemniscal tonotopic pathway

Auditory information ascending from the cochlea and lower brainstem centers must first be transmitted through the MGB before being subsequently conveyed to auditory cortical areas (Winer, 1984; Sherman and Guillery, 2006; Jones, 2007). The functional organization of the auditory TC pathways is determined in part by their thalamic nuclear origins and cortical areal targets (Kaas and Hackett, 2000; Lee and Winer, 2011). Within the thalamus, the MGB is divided into three main subdivisions, i.e., the ventral division, the dorsal division, and the medial division, delineated on the basis of their connections, cytoarchitecture, and physiological properties (Winer, 1984; Imig and Morel, 1985; Huang and Winer, 2000; de la Mothe et al., 2006; Lee and Winer, 2008). Similarly, the auditory cortex can be distinguished into “core”, “belt”, and “parabelt” areas (Kaas and Hackett, 2000; Lee and Winer, 2008). The neuroanatomical pathways connecting these thalamic nuclei to the auditory cortical areas constrain the functional transformations occurring at this stage of auditory processing (Winer et al., 2005).

The classically described tonotopic arrangement of frequency is established through topographic projections originating in the auditory periphery and is a fundamental organizing principle of the lemniscal TC pathway from the ventral division of the MGB (MGBv) to the “core” auditory cortical fields (Brandner and Redies, 1990; Lee and Winer, 2005, 2008). Within the auditory thalamus, the MGBv is the main division with a tonotopic organization (Calford, 1983; Imig and Morel, 1983; Hackett et al., 2011). Neurons in the MGBv mainly project to layers 3 and 4, as well as branching to other supragranular and infragranular layers in A1 (Huang and Winer, 2000; Broicher et al., 2010; Smith et al., 2012; Lee and Imaizumi, 2013; Saldeitis et al., 2014). Neurons in the MGBv have smaller cell bodies and are arranged in laminar rostrocaudal sheets, with their dendritic fields aligned in parallel along the sheet (Winer, 1992). The neurons in each sheet respond to similar sound frequencies (Imig and Morel, 1985), which establishes the observed tonotopic organization. Orientation of these sheets differs among species, dorsoventrally in the rat and lateromedially in the mouse and cat (Imig and Morel, 1983; Hackett et al., 2011; Storace et al., 2011). In comparison, the dorsal and the medial divisions of the MGB (MGBd and MGBm) are anisotropically organized (Winer, 1992), resulting in no tonotopic organization (Calford, 1983). Neurons in these nuclei have broader, multipeaked, complex, and/or multimodal receptive fields, in contrast to the sharply tuned neurons of the MGBv (Calford, 1983; Anderson et al., 2007; Anderson and Linden, 2011). The MGBm, in particular, contains some of the largest cell bodies in the MGB, and is further distinguished by a sparser packing of cell bodies (Winer, 1992). The TC projections of the MGBd largely targets “belt” auditory cortical fields, such as the secondary auditory cortex, terminating primarily in layers 3 and 4, while TC neurons of the MGBm project more broadly across many auditory cortical areas, primarily targeting layer 1 in each area (Huang and Winer, 2000; Kaas and Hackett, 2000; Lee and Winer, 2008; Smith et al., 2012). The core auditory cortical fields are defined by their tonotopic organization and their TC inputs arising from the MGBv (Kaas, 2011). The number of core auditory cortical fields with tonotopic organization differs among species: two (and possibly more) core fields, the primary auditory cortex (A1) and the anterior auditory field (AAF) (and possibly the posterior and the ventroposterior fields), in carnivores and rodents and three core fields, the rostrotemporal field, the rostral field, and A1, in primates, possibly including humans (Kaas, 2011). Among these fields, A1 is common to all mammalian species studied thus far and is the most extensively studied field. Consequently, we will focus on sound frequency transformation from MGBv to A1 in the lemniscal pathway.

**Figure 1:**
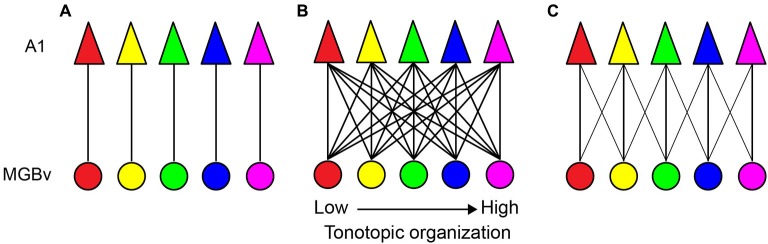
**Sound frequency thalamocortical transformation models. (A)** A point-to-point model. Each circle and triangle illustrates a cluster of neurons tuned to a similar characteristic frequency (CF) in the MGBv and A1, respectively. **(B)** A widespread divergent and convergent model. All neuron clusters are heavily connected. **(C)** A constrained divergent and convergent model. A cluster of A1 neurons receives convergent thalamocortical inputs from sound frequencies within 1/3 octave of different best frequencies.

In this respect, several TC models have been proposed to describe the functional organization of auditory lemniscal projections (Figure 1; Brandner and Redies, 1990; Molinari et al., 1995; Miller et al., 2001; Lee and Winer, 2005). Among these possible models, topographic projections in the lemniscal pathway may be organized in a point-to-point manner, i.e., limited divergence from a cluster of thalamic neurons to limited areas of A1 (Figure 1A; Brandner and Redies, 1990). Based on a linear estimation of spectro-temporal receptive fields (STRFs) by simultaneous single-unit recordings from functionally connected MGBv and A1 neurons in ketamine-anesthetized cats, this suggestive point-to-point TC organization may contribute to direct inheritance of STRFs from the MGBv to A1, but is not the major organization type (Miller et al., 2001). Rather, STRFs are created by convergence in the TC transformation, i.e., thalamic neurons with partially overlapped STRFs converge on single A1 neurons. Anatomical studies have supported this view. Anterograde tracer injections in the MGB or retrograde tracer injections in A1 clearly demonstrate divergent or convergent projections, respectively, in rodents, carnivores, and primates (Figure 1B; Molinari et al., 1995; Huang and Winer, 2000; Lee et al., 2004b; Lee and Winer, 2008; Read et al., 2008; Razak and Fuzessery, 2010; Hackett et al., 2011; Storace et al., 2011). This divergent and convergent model also suggests that tonotopy or characteristic frequency (CF) of each cortical neuron or a cluster of neurons results from computational processing. Furthermore, this divergent and convergent model is constrained by a general rule for sound frequency transformations, at least, in the cat. For secure functional transformations, the MGBv and A1 neurons require an alignment of less than 1/3 octave difference in best frequency (sound frequency evoked best response in a neuron at a given sound level) (Miller et al., 2001). To fully activate an A1 neuron, synaptic convergence from 20–25 MGBv neurons is required. However, as noted by Miller et al. (2001), these rules might be biased toward the most robust and strongest TC connectivity. Nevertheless, such divergence and convergence is constrained, rather than profligate and widespread, originating from circumscribed thalamic areas representing sound frequencies within a 1/3 octave domain, as illustrated in Figure 1C.

## Spectral receptive field obtained by classical approaches

Sound frequency transformations can be characterized through analyses of the spectral receptive field (SRF). The SRF is, in general, measured based on a frequency-threshold tuning curve (response to sound level as a function of sound frequency). A common measure is the *Q*-factor by which CF is divided by a linear measure of bandwidth at a given sound level above threshold (e.g., *Q*10; *Q*-value at 10 dB above threshold) (Imaizumi and Schreiner, 2007). Because the *Q*-value is a normalized measure, the larger the *Q*-value, the more sharply tuned are the neurons.

**Figure 2:**
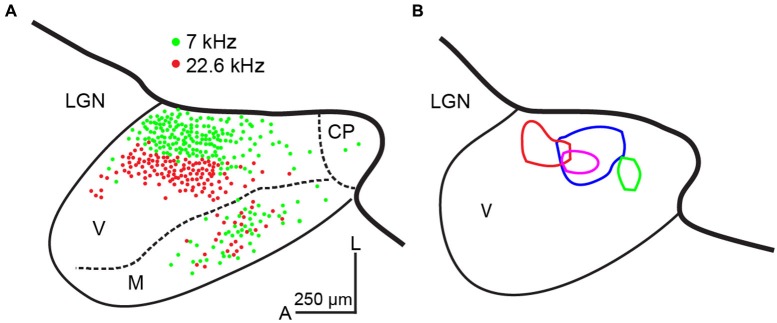
**Convergence from thalamocortical projections in the mouse. (A)** Double retrograde tracer injections in physiologically identified locations (7 and 22.6 kHz) in mouse A1. Labeled neurons in a semi-horizontal section (15° from the horizontal plane) of the MGB are plotted. These examples illustrate convergence to a cluster of A1 neurons. Modified from Hackett et al. (2011) with permission. **(B)** Four activated thalamic maps (different colors) in a semi-horizontal section (15° from the horizontal plane) of the MGB by laser-scanning photostimulation while making whole-cell recordings from layer 4 A1 neurons in the mouse. These examples illustrate convergence to single A1 neurons. CP: caudal pole, LGN: the lateral geniculate nucleus, M: medial division of the MGB, V: ventral division of the MGB, A: anterior, L: lateral. The scale bar in **(A)** is applied to **(B)**. To adjust scale of fixed tissue to live tissue, a factor of 1.4 is applied. Unpublished data.

Neurons in the MGBv are, in general, more sharply tuned than in non-lemniscal divisions of the MGB (Rouiller et al., 1981; Calford, 1983; Edeline et al., 1999; Anderson et al., 2007; Anderson and Linden, 2011). However, no clear spatial organization of thalamic SRFs is available, due to the deep location in the brain. On the other hand, spatial organization of SRFs in A1 is available in many species. These transformations at the TC synapse are mediated by the excitatory neurotransmitter, glutamate, from the MGBv to A1 (Lee, 2014). Thus, only the excitatory SRF is directly transformed through TC projections. Based on the divergent and convergent TC model (Figure 1C), SRFs in A1 neurons can be spatially homogeneous. This is seen in rodent A1 based on multi-unit recordings from layer 4 under pentobarbital or ketamine anesthesia (Polley et al., 2007; Guo et al., 2012). Anatomical studies employing retrograde tracer injections in the mouse and rat A1 support this idea by demonstrating wide convergent TC projections (Figure 2A; Polley et al., 2007; Hackett et al., 2011; Storace et al., 2011). Furthermore, the CF gradient (CF distance divided by cortical surface distance) is linear (Hackett et al., 2011; Storace et al., 2011). Regarding the thalamus, neurons in the MGBv are, in general, sharply tuned to CF, although only a handful of studies have quantified SRFs in the MGBv (Calford, 1983; Bordi and LeDoux, 1992; Edeline et al., 1999; Anderson and Linden, 2011; Bartlett et al., 2011). Based on the assumption of sharply tuned MGBv neurons and using local field potentials that record subthreshold activity in rat A1, Kaur et al. (2004, 2005) proposed a classical convergent TC model, in which broadly-tuned neurons in A1 are constructed from sharply-tuned neurons in the MGBv that merge with intracortical sideband input. Thus, TC transformations of SRFs depend on thalamic and intracortical sources. The relative functional weights of these convergent inputs are of some debates (de la Rocha et al., 2008) and is being addressed using modern experimental approaches, as discussed below.

There are a few clear cases of non-homogenous distribution of SRFs in A1 (reviewed in Imaizumi and Schreiner, 2007). Among these, the most well documented example is found in A1 of the echolocating mustached bat (Suga, 1994). A large area in A1, called the Doppler-shifted constant frequency (DSCF) area, is devoted to a particular sound frequency range (60.6–62.3 kHz) for their ethological and ecological behaviors. A1 neurons in the DSCF area are extremely sharply tuned to CF; *Q*50 values range from ∼10 to 500 or higher (Suga and Manabe, 1982). Neurons in the anterior and posterior parts of the DSCF area are more broadly tuned in A1. DSCF neurons are also found in the MGBv. Based on single-unit recordings in the awake state, SRFs in DSCF neurons become sharper significantly through TC transformation due to the stronger cortical inhibitory innervation (Suga et al., 1997). This type of sharpening of SRFs through TC transformation is not usually found in rodent A1, as described above. Thus, behaviorally important sound frequency information may be further filtered intracortically beyond the TC transformations.

**Figure 3:**
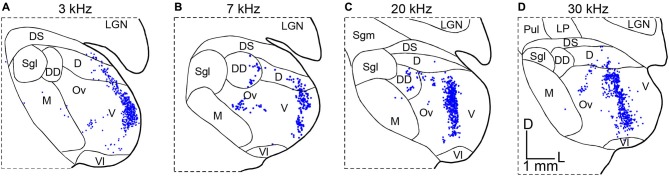
**Convergence from thalamocortical projections in the cat.** Retrograde tracers were injected along physiologically identified isofrequency lamina in cat A1. The panels illustrate the resulting patterns of retrogradely labeled neurons in coronal sections of the MGB in four different experiments. **(A)** Labeling in the MGB following injections in the 3 kHz CF lamina of A1. **(B)** Labeling in the MGB following injections in the 7 kHz CF lamina of A1. **(C)** Labeling in the MGB following injections in the 20 kHz CF lamina of A1. **(D)** Labeling in the MGB following injections in the 30 kHz CF lamina of A1. Modified from Lee et al. (2004b). D: dorsal nucleus of the MGB, DD: deep dorsal nucleus of the MGB, DS: dorsal superficial nucleus of the MGB, LGN: lateral geniculate nucleus, LP: lateroposterior thalamic nucleus, M: medial division of the MGB, Ov: *pars ovoidea* of the MGB, Pul: pulvinar, Sgl: suprageniculate nucleus, lateral part, Sgm: suprageniculate nucleus, medial part, V: ventral division of the MGB, Vl: ventrolateral nucleus of the MGB, D: dorsal, L: lateral. The scale bar in **(D)** is applied to all panels.

Another example of non-homogenous distribution of SRFs is cat A1 (Schreiner et al., 2000; Read et al., 2001; Imaizumi and Schreiner, 2007). Based on multi-unit recordings from cortical layers 3b and 4 in ketamine anesthetized state, broadly- and sharply-tuned neuron clusters based on *Q*40 values are alternately located dorsoventrally perpendicular to the tonotopic frequency axis. Unlike DSCF neurons in the mustached bat A1, *Q*40 values in the cat A1 are not very high. However, the spatial transition between broadly- and sharply-tuned neuron clusters is obvious. This modular functional organization of broad and sharp SRFs is found only between the 5 and 20 kHz mid-frequency areas (Imaizumi and Schreiner, 2007). Furthermore, CF gradients in the cat A1 are not linear, exhibiting steeper gradients in the low- to mid-frequency areas (<10 kHz), flat between the 10 and 20 kHz, and shallower gradients in the high-frequency areas (>20 kHz), which differs from the more linear CF gradients in the periphery (Greenwood, 1990). A natural question arises as to whether these physiological transformations of SRFs are supported by alterations to the TC connections. Anatomical studies addressing this issue used a retrograde tracer injected at two or three different locations along four different frequency contours in A1. The resultant pattern of thalamic labeling in the MGB is similar and independent of the frequency contours injected, which indicates that the relative convergence of TC projections is similar across all frequency ranges (Figure 3; Lee et al., 2004b). This suggests similar TC transformation across different frequency areas (e.g., Figure 1C). Interestingly, the distribution of labeled neurons in the cat MGBv is proportionally smaller than that observed in the rodent (Figures 2A, 3), suggesting wider proportional convergence of thalamic inputs in rodents. This view is supported by related anatomical studies that focus on physiologically identified micro-domains in the cat A1. Read et al. (2008) made finer targeted retrograde tracer injection in sharply-tuned neuron clusters in different isofrequency axes of cat A1 and found similar distributions of labeled neurons in the MGBv as those shown in Figure 3. Thus, the modular functional organization of SRFs and different CF gradients may be created within A1 through the TC transformation.

## Modern experimental approaches

The examples discussed above employed classical experimental techniques. However, recent studies of the TC transformation are employing more modern experimental techniques, such as two-photon calcium imaging, whole-cell recordings, optogenetics, and photostimulation methods. These studies tend to focus on the mouse and rat A1 due to their cost and empirical tractability with these new approaches. How have these modern approaches updated our knowledge?

## *In vivo* two-photon calcium imaging

The advent of modern approaches using *in vivo* two-photon calcium imaging enables us to measure physiological properties and to identify anatomical locations simultaneously (Ohki et al., 2005). This powerful experimental application is particularly useful for studying the neocortex. Due to limitation of the laser reaching the deeper layers of the auditory cortex, researchers originally focused on studying tonotopy of single neurons in the supragranular layers 2 and 3 of the mouse A1 (Bandyopadhyay et al., 2010; Rothschild et al., 2010). These two studies employed different stimulation and analytical methods: Bandyopadhyay et al. (2010) employed amplitude-modulated pure tones and best frequency in calcium responses, while Rothschild et al. (2010) employed pure tone pips (same as classical approaches) and extracted frequency-threshold tuning curves in spike counts from calcium responses using deconvolution techniques (Yaksi and Friedrich, 2006). They observed highly heterogeneous populations of single neurons at a finer scale and presence of tonotopy on a large scale. These heterogeneous populations of single neurons in layers 2 and 3 may also correlate with heterogeneous tuning to sound frequencies in neighboring dendritic spines of the same layers (Chen et al., 2011). An important question arises here: is this phenomenon specific to supragranular layers in the mouse A1? Polley and his coworkers found the presence of tonotopic organization with high-resolution cortical mapping in layer 4 of the mouse A1 (Hackett et al., 2011). Later, Winkowski and Kanold (2013) identified heterogeneous populations of single neurons in a cluster of layers 2 and 3 and homogenous populations in layer 4 using *in vivo* two-photon calcium imaging (Winkowski and Kanold, 2013), in agreement with the classical approaches using multi-unit extracellular recordings and high-resolution cortical mapping studies of layer 4. These findings align with the notion that, as discussed below, a cluster of neurons in layer 4 receive the same degree of frequency convergence as single neurons in mouse TC transformation. Thus, the mouse A1 preserves tonotopic organization at the single neuron level in layer 4.

## *In vivo* whole cell recordings

One important contribution of these modern approaches is towards isolating excitatory inputs from inhibitory inputs in the TC transformation. For instance, recent application of *in vivo* whole-cell recordings in A1 has enabled an examination of the excitatory and inhibitory responses to external sound stimulus by holding the neuron at different membrane potentials close to the reversal potentials of the relevant ions. As stated above, because TC projections carry only excitatory inputs to A1, the inhibitory inputs are deduced to originate from cortical circuits within A1. An important question here is to what extent TC pathways carry excitatory input to A1 regarding frequency integration range. As discussed above, despite the wide anatomical convergence from MGBv neurons (Figures 1, 2), is TC excitatory input still very narrow (Kaur et al., 2004, 2005)? One approach to isolating TC input from cortical input is to apply muscimol locally in A1. A problem with this approach is that muscimol also activates GABA_B_ receptors on TC axons (Yamauchi et al., 2000). This issue can be circumvented by inactivating these receptors by a GABA_B_ receptor antagonist, SCH50911, application (Liu et al., 2007). In this study, Liu et al. (2007) applied a cocktail of muscimol and SCH50911 in whole-cell recordings from TC recipient A1 neurons in the rat, and found that TC input results in smaller response amplitude and broader frequency convergence in A1 neurons (Liu et al., 2007).

Such pharmacological approaches have become refined through the advent of optogenetic methods that enable the specific activation or inactivation of particular neuronal cell types, such as the different classes of cortical inhibitory neurons (Taniguchi et al., 2011; Madisen et al., 2012). In a more recent study using whole-cell recordings combined with optogenetic activation of parvalbumin-expressing inhibitory neurons in the mouse A1 (the low- to mid-frequency areas), the same group (Zhang and his colleagues) revealed different results compared to their pharmacological studies: tuning bandwidth in A1 neurons is similar before and after optogenetic applications (Li et al., 2013). This discrepancy may result from differences in the selectivity of these approaches; cortical inhibition is broadly affected with pharmacological approaches, while the optogenetic approach selectively affected parvalbumin-expressing inhibitory neurons. Alternatively, the discrepancy may result from differences in the effective synaptic sites: a cocktail of muscimol and SCH50911 affects pre- and postsynaptic sites, while optogenetic applications affect only presynaptic neurons.

## Laser-scanning photostimulation in *in vitro* thalamocortical slices

**Figure 4:**
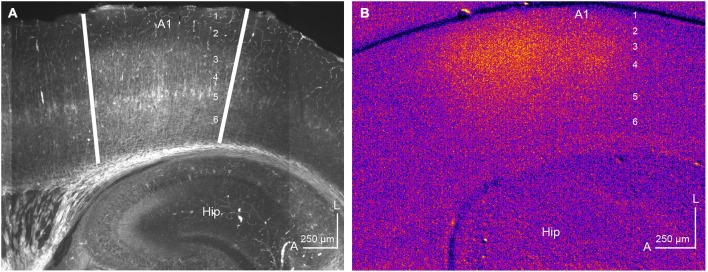
**Identification of A1 by immunohistochemistry and flavoprotein autofluorescent imaging. (A)** SMI-32 is expressed well in layers 3 and 5 in a semi-horizontal thalamocortical section (50 μm) of mouse A1. The anterior and posterior A1 borders are illustrated by white lines. Unpublished data. **(B)** Flavoprotein autofluorescent imaging in the semi-horizontal thalamocortical section (500 μm) of mouse A1. A rectangle positive current (200 μA) was injected for one second in the MGBv using a concentric bipolar microelectrode. Hot colors show activated areas, corresponding to A1. The numbers in A1 indicate cortical layers. Hip: hippocampus, A: anterior, L: lateral. Modified from Lee and Imaizumi (2013).

Traditionally, the auditory TC system has been studied using *in vivo* techniques, such as those discussed above. However, advances in our understanding of TC microcircuits have emerged from investigations employing *in vitro* rodent brain slice preparations preserving intact TC connectivity (Cruikshank et al., 2002; Kotak et al., 2005; de la Rocha et al., 2008; Lee and Sherman, 2008). One persistent issue with the use of these *in vitro* slices is to establish A1 borders in a TC preparation for whole-cell recording locations in the slice relative to *in vivo* physiological parameters, such as CF. Different groups have reported slightly different A1 locations in the TC preparations presumably due to differences in blocking (Cruikshank et al., 2002; Broicher et al., 2010; Oviedo et al., 2010; Lee and Imaizumi, 2013). Our approach to identifying the A1 borders is with cytoarchitectonic markers, in particular, immunoreactivity to SMI-32 in resectioned slices from TC preparations. SMI-32 antibody recognizes neurofilaments in pyramidal neurons in layers 3, 5, and 6 and its expression pattern is cortical area-specific (Sternberger and Sternberger, 1983; Mellott et al., 2010). Figure 4A illustrates a representative example of SMI-32 immunoreactivity in A1 of the mouse TC slice preparation. SMI-32 is expressed well in layers 3 and 5 of A1, but not in adjacent anterior or posterior areas, which clearly delineates the anatomical borders (illustrated by white lines). Unfortunately, the underlying mechanisms of area-specific expression patterns of SMI-32 are not known. These borders align roughly with the anterior high-frequency border (above the rostral edge of the hippocampus) and the posterior low-frequency border (roughly the mid-point of the hippocampus) (Figure 4A). These A1 borders also correlate with borders established using an *in vitro* physiological measure, flavoprotein autofluorescent imaging (Llano et al., 2009; Lee and Imaizumi, 2013). Flavoprotein autofluorescent imaging is a non-hemodynamic measure of neuronal metabolism, which has been employed both *in vivo* and *in vitro* to examine broad spatial patterns of neuronal activity (Shibuki et al., 2003; Takahashi et al., 2006; Llano et al., 2009; Lee and Imaizumi, 2013). Using this *in vitro* approach, we have found that the A1 borders established by SMI-32 immunoreactivity (Figure 4A) align well with the spatial activation pattern observed using *in vitro* flavoprotein autofluorescent imaging, following electrical stimulation of the MGBv (Figure 4B; Lee and Imaizumi, 2013). The area adjacent to A1, putatively AAF, is not activated by electrical stimulation of the MGBv because the anatomical connections to AAF are unlikely preserved in our TC blocking.

Using these criteria to identify A1 in the *in vitro* slice preparation, we examined whether the spatial pattern of TC convergence onto a single recorded A1 neuron varies from the degree of convergence obtained by classic retrograde tracer injection studies discussed above (Figures 2A, 3). These studies take advantage of the accessibility of structures in the slice preparation and utilize a method of functional mapping known as laser-scanning photostimulation via uncaging of glutamate (Callaway, 1994; Shepherd et al., 2003; Lee and Sherman, 2008). In brief, caged glutamate applied to the bath can be activated focally using a galvo-positioned laser beam, which results in stimulation of a narrow column of neurons (∼50 μm) in the slice (Lam and Sherman, 2005; Shepherd, 2012; Lee et al., 2013). Using this approach, the extent of functional convergence of thalamic input to single neurons in layer 4 of A1 can be directly assessed. As examples, the thalamic regions eliciting excitatory responses in layer 4 A1 neurons recorded using whole-cell patch clamp are illustrated by contours in four representative cases (Figure 2B). We compared these thalamic regions of single-cell convergence with the regions of convergence in the MGB from physiologically targeted injections demonstrated by Hackett et al. (2011), by aligning our maps with their labeling patterns (thicker black lines in Figure 2; estimated diameter of their tracer injections is ∼250 μm). To compensate for shrinkage of tissues by fixation (e.g., 30%), we applied a factor of 1.4 to scale the results from fixed TC semi-horizontal section to our results obtained from the live *in vitro* preparations. While the distribution of labeled neurons in the MGBv (Hackett et al., 2011; Figure 2A), corresponding to frequency convergence to a cluster of A1 neurons (e.g., ∼250 μm radius), appears similar to the thalamic activated map, corresponding to frequency convergence to an A1 neuron (Figure 2B), the thalamic activated map originates from more restricted areas than those identified by retrograde labeling. However, one important point in distinction is the absence of responses from photostimulation in the medial division of the MGB (Figure 2B), which is relatively common in retrograde tracer injections in A1 (Lee et al., 2004b; Read et al., 2008; Hackett et al., 2011; Storace et al., 2011). This discrepancy is largely attributable to the injected tracers spreading into layer 1, which is the primary target of projections from the medial division (Huang and Winer, 2000). This comparison of convergence to single neurons and a cluster of neurons in A1 suggests that the frequency range of thalamic inputs aligns with the functional limits proposed by Miller et al. (2001). However, single layer 4 A1 neurons receive TC input from restricted areas within a frequency lamina in the MGBv that are encompassed by the regions identified from the relatively larger retrograde injections.

## *In vitro* imaging techniques in thalamocortical slices

A powerful application of *in vitro* TC slice preparations has been their use in mapping synaptic input sites on the dendrites of TC recipient A1 neurons. Richardson et al. (2009) filled A1 neurons in layers 3 and 4 with Alexa 594 for cell morphology and a calcium indicator, Fluo-5F, through the whole-cell recording pipette. They employed electrical stimulation on TC fibers and measured calcium response in dendritic spines and the shaft using two-photon calcium imaging. TC recipient A1 neurons in layers 3 and 4 preferentially receive synaptic input on stubby dendrites from TC fibers close to the soma (Richardson et al., 2009). Furthermore, the distribution of these dendritic spines is similar to those that receive input from intracortical layer 4.

Although *in vitro* two-photon calcium imaging provides high spatial resolution (e.g., single cell bodies and dendritic spines), this technique often sacrifices temporal resolution. Imaging using voltage sensitive dyes allows experimenters to assess responses with higher temporal resolution to external stimulus, although spatial resolution is sacrificed (Grinvald and Hildesheim, 2004). Broicher et al. (2010) applied this technique to *in vitro* TC slice preparations. Their TC slice preparations differ from those more typically used. In particular, the blocking angle in their preparation is 25° from horizontal axis, while most, including ourselves, employ the 15° angle based on Cruikshank et al. (2002). Their A1 borders, defined using SMI-31, extended to more anterior cortical regions than we have observed (Figure 4A). Furthermore, their thalamic electrical stimulation often activated larger areas outside A1, while our thalamic electrical stimulation activated only A1 (Figure 4B; Lee and Imaizumi, 2013). Their *in vitro* voltage sensitive dye imaging shows unexpected results. Supragranular layers had the shortest latencies and largest response amplitude when thalamic electrical stimulation activated widespread cortical areas including adjacent auditory cortical fields. However, when thalamic electrical stimulation activated only A1, the granular layer was found to have the shortest latencies (Broicher et al., 2010). These results can not be accounted for by anatomical studies (Huang and Winer, 2000; Smith et al., 2012; Saldeitis et al., 2014), in which supragranular layers do not receive extensive TC input, or *in vivo* electrophysiological experiments using multi-channel silicon probes in cat A1, in which layers 4 and 6 show the shortest latencies to external sound stimulus (Atencio and Schreiner, 2010). Polysynaptic input from other cortical layers, such as layer 4 (Barbour and Callaway, 2008), also does not account for such short latencies. The apparent mismatch between the results obtained from these *in vitro* voltage sensitive dye studies and those obtained using classical methods would benefit from future investigation.

## Future directions

The auditory system has evolved and adapted to each animal’s environment and behavioral requirements (Imaizumi and Lee, 2013). For example, rodents have to detect and avoid predators, such as cats, before nocturnal hunters detect them or vice versa for survival. Thus, different animal species have unique variations on neural processing pathways and the overall pattern of TC organization, ranging from the relatively homogenous organization in rodent A1 to the non-homogenous organization in the mustached bat and cat A1 (Kaas, 2011), which parallels organization of other sensory systems (Catania, 2012). These suggest that results or theory obtained from one species can not be simply applied to other species. Current research trends have shifted to rodent models, in particular the mouse, largely due to the cost and the genetic tractability of this system (Jones et al., 2009; Madisen et al., 2012). Although a comparative approach is still warranted, using the mouse model system, one goal may be to determine the detailed synaptic organization on all possible synaptic sites in excitatory and inhibitory neurons in the different cortical layers and how auditory information is computed within single neurons, clusters of neurons, and across A1. Although daunting, such a systematic and detailed exploration of auditory TC circuitry provides a target for future exploration.

## Conflict of interest statement

The authors declare that the research was conducted in the absence of any commercial or financial relationships that could be construed as a potential conflict of interest.
